# Dendritic Cell-Based Immunotherapy in Multiple Myeloma: Challenges, Opportunities, and Future Directions

**DOI:** 10.3390/ijms23020904

**Published:** 2022-01-14

**Authors:** Emma Verheye, Jesús Bravo Melgar, Sofie Deschoemaeker, Geert Raes, Anke Maes, Elke De Bruyne, Eline Menu, Karin Vanderkerken, Damya Laoui, Kim De Veirman

**Affiliations:** 1 Laboratory of Hematology and Immunology, Myeloma Center Brussels, Vrije Universiteit Brussel, 1090 Brussel, Belgium; emma.mariette.verheye@vub.be (E.V.); anke.maes@vub.be (A.M.); elke.de.bruyne@vub.be (E.D.B.); eline.menu@vub.be (E.M.); karin.vanderkerken@vub.be (K.V.); 2 Laboratory of Myeloid Cell Immunology, VIB Center for Inflammation Research, 1050 Brussels, Belgium; jesus.bravo.melgar@vub.be (J.B.M.); sofie.deschoemaeker@vub.be (S.D.); geert.raes@vub.be (G.R.); 3 Laboratory of Cellular and Molecular Immunology, Vrije Universiteit Brussel, 1050 Brussels, Belgium

**Keywords:** multiple myeloma, dendritic cells, immunotherapy, vaccination

## Abstract

Immunotherapeutic approaches, including adoptive cell therapy, revolutionized treatment in multiple myeloma (MM). As dendritic cells (DCs) are professional antigen-presenting cells and key initiators of tumor-specific immune responses, DC-based immunotherapy represents an attractive therapeutic approach in cancer. The past years, various DC-based approaches, using particularly ex-vivo-generated monocyte-derived DCs, have been tested in preclinical and clinical MM studies. However, long-term and durable responses in MM patients were limited, potentially attributed to the source of monocyte-derived DCs and the immunosuppressive bone marrow microenvironment. In this review, we briefly summarize the DC development in the bone marrow niche and the phenotypical and functional characteristics of the major DC subsets. We address the known DC deficiencies in MM and give an overview of the DC-based vaccination protocols that were tested in MM patients. Lastly, we also provide strategies to improve the efficacy of DC vaccines using new, improved DC-based approaches and combination therapies for MM patients.

## 1. Introduction

Multiple myeloma (MM) is the second most common hematological malignancy. Due to the uncontrolled proliferation and subsequent accumulation of a single clone of terminally differentiated B cells in the bone marrow (BM) niche, advanced stages of MM are accompanied by clinical features, such as anemia, renal failure, and hypercalcemia in conjunction with osteolytic bone lesions [1]. MM cells secrete a monoclonal immunoglobulin (M-protein) with specific antigenic determinants, the idiotype (Id), which can be considered as a tumor-associated antigen [2]. MM evolves from an asymptomatic premalignant stage termed monoclonal gammopathy of undetermined significance (MGUS) that can progress into smoldering MM (SMM) and, lastly, into active/symptomatic MM [3]. Patients are primarily diagnosed at the age of 65–70 years; however, 37% are younger than 65 years, and the majority of patients eventually succumb to progressive disease (PD) with a median life expectancy of 5–7 years [4,5].

High-dose chemotherapy followed by autologous hematopoietic stem-cell transplantation (auto-HSCT) is considered the standard of care for the younger, more fit MM patients (less than 70 years of age). For transplant-ineligible MM patients, the treatment has evolved significantly over the last decades from alkylating agents and steroids to triple and quadruple combinations including proteasome inhibitors (e.g., bortezomib, carfilzomib) and immunomodulatory drugs (e.g., lenalidomide) [6,7]. The past years, immunotherapy has opened a new era in the treatment of MM [8]. Monoclonal antibodies, bispecific antibodies, immune checkpoint inhibitors, vaccines, and adoptive T cell therapies have been actively investigated, and some of them achieved remarkable clinical successes in MM patients [8]. The monoclonal CD38-targeting antibodies daratumumab or isatuximab, CS1-targeting elotuzumab, and the B-cell maturation antigen (BCMA)-targeting antibody drug conjugate belantamab mafodotin are approved drugs for the treatment of MM [8,9,10]. In 2021, the Food and Drug Administration (FDA) approved abecma (idecabtagene vicleucel), the first chimeric antigen receptor (CAR) T cell therapy for MM patients [11,12]. Despite the advances in cancer immunotherapy, the majority of patients still relapse, and responses are hampered by immune escape mechanisms and the presence of an immunosuppressive BM microenvironment.

Immune dysfunction and immunosuppression are both hallmarks of MM disease [13,14,15]. Common immunodeficiencies include (a) dysfunctional CD8^+^ T cells and natural killer (NK) cells [16], (b) the imbalanced production of immunogenic and tolerogenic cytokines [17,18], (c) upregulated inhibitory immune checkpoints [19], (d) impaired antigen presentation capacity [20,21], and (e) infiltration of immunosuppressive cells in the BM niche. Myeloid-derived suppressor cells (MDSCs), tumor-associated macrophages (TAMs), regulatory T cells (Tregs), T helper (Th) 17 cells, and mesenchymal stromal cells are major cellular components of this suppressive microenvironment [14,22,23,24] and contribute to MM cell survival and drug resistance.

The use of dendritic cells (DCs) as a platform for cancer vaccine development is another safe and promising strategy to enhance tumor-specific host immune responses [25,26]. DCs are indispensable for an adequate immune response, as they bridge the innate and the adaptive immune system in response to different pathogens, viruses, bacteria, and even tumor cells. As master antigen-presenting cells (APCs) present in all tissues, DCs capture, process, and present tumor-(neo)antigens to naïve T cells via major histocompatibility complex (MHC) molecules. Depending on the antigen (Ag) nature, with MHC-I presenting endogenous Ags and MHC-II presenting exogenous Ags, DCs elicit adaptive CD8^+^ or CD4^+^ T cell immune responses, respectively [27]. Due to their capacity for cross-presenting Ags and inducing specific cellular (T cells) and humoral (B cells) immune responses, DCs are a promising tool for immunotherapy in MM. However, in the context of cancer, it is important to note that DCs can also exert immunosuppressive and pro-tumoral effects either by an increased expression of immune checkpoints or by the elaboration of tolerogenic signals. On the other hand, DCs can also themselves be suppressed, for instance, by tumor-derived cytokines and immunosuppressive cells, such as Tregs [28,29,30].

Over the past decades, many DC-based therapies have been clinically tested in MM; however, long-term clinical responses were rather inconsistent [31,32]. In this review, we briefly discuss the development as well as the phenotypical and functional characteristics of the major DC subsets in the BM. Further details concerning the challenges, the opportunities, and the future directions of DC-based immunotherapy in MM are addressed focusing on the DC deficiencies in the BM niche, the currently used DC-based vaccination strategies in clinical trials, and the therapeutic potential of new, improved DC-based approaches, and combination therapies for MM patients.

## 2. Dendritic cell (DC) origin and Function in Healthy Tissue

### 2.1. Development of DCs in the BM Niche

DCs, named after the Greek word “dendron” because of their tree-like morphology, were first documented by Steinman and Cohn in 1973. Of note, this discovery was awarded the 2011 Nobel Prize in Physiology or Medicine [33].

Over the last few decades, great attention has been given to DC biology in healthy and malignant tissue. DC characterization reveals a heterogeneous cell population, with each subset having its specific lineage, markers, location, migratory, and functional capacity. An ongoing debate concerns the DC classification system and nomenclature, as it is a difficult and controversial subject. Guilliams et al. proposed a nomenclature for mononuclear phagocytes by classifying cells primarily according to the ontogeny and secondary by the location, function, and phenotype [34]. Currently, two ontogenically distinct DC subsets are described: classical/conventional DCs (cDCs) and plasmacytoid DCs (pDCs). Furthermore, an additional subset including monocyte-derived cells also referred to as monocyte-derived DCs (moDCs) is worth mentioning. However, there is some disagreement among researchers as to whether to classify these cells as macrophages or as DCs [34,35,36].

Even though the DC subsets differ in several aspects, they all arise from a multipotent hematopoietic stem cell (HSC; CD34^+^) located in the BM in a stepwise differentiation process called “hematopoiesis”. Due to lineage-specific nuclear transcription regulatory factors, these HSC differentiate into the desired cell type and the specific subtypes. First, the HSCs develop into two different cell lineages: common lymphoid progenitor (CLP) and common myeloid progenitor (CMP) cells [37]. It is the latter that has generally been acknowledged to give rise to the different DCs subsets since the CMP cells differentiate into granulocyte macrophage DC precursors (GMDP), with the prospect to develop granulocyte, macrophage and DC populations. Subsequently, GMDPs differentiate into macrophage DC progenitors (MDP) and common DC progenitors (CDPs) with the capacity to give rise to pre-cDCs and pre-pDCs. While pre-pDCs produce pDCs in the BM, pre-cDCs are already committed to the cDC1 and cDC2 lineage in the BM niche (termed pre-cDC1 and pre-cDC2) [38,39,40]. Following this differentiation process, pre-cDCs and pDCs leave the BM and enter the circulation where the pre-cDCs differentiate into fully functioning cDC1 and cDC2 [41,42,43]. Although this suggests that cDCs and pDCs derive from CMPs, other studies suggest that pDCs arise from other precursors, namely CLPs [39,44,45,46]. In addition, moDCs differentiate from blood circulating monocytes in inflammatory conditions.

**Figure 1:**
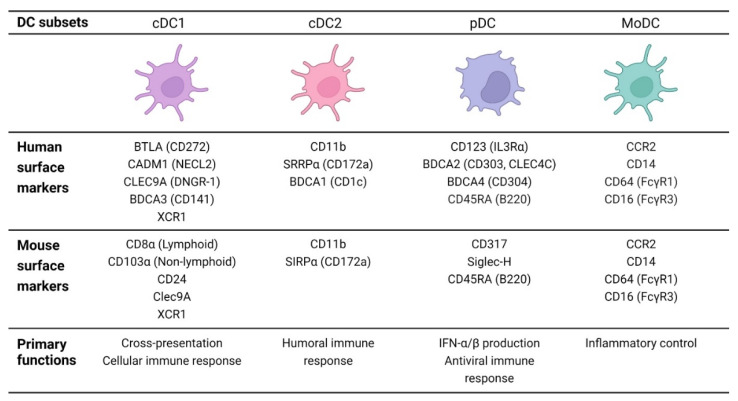
The distinct DC subsets with their respective surface markers (human and mouse) and primary functions. Markers can vary across tissues and depend on the physiological setting. cDC1, type 1 classical/conventional dendritic cell; cDC2, type 2 classical/conventional dendritic cell; pDC, plasmacytoid dendritic cell; moDC, monocyte-derived dendritic cell; BTLA, B and T cell lymphocyte attenuator; CADM1, cell adhesion molecule 1; CLEC9A, C-type-lectin 9A; BDCA3, blood dendritic cell antigen 3; XCR1, XC chemokine receptor 1; CLEC10A, C-type-lectin 10A; BDCA1, blood dendritic cell antigen 1; IL3Rα, interleukin 3Rα; BDCA2, blood dendritic cell antigen 2; CLEC4C, C-type-lectin C4; BDCA4, blood dendritic cell antigen 4; Siglec-H, sialic acid-binding immunoglobulin-like lectin-H; CCR2, C-C motif chemokine receptor 2; FcγR1, Fc-gamma receptor 1; FcγR3, Fc-gamma receptor 3.

DC development is strongly cytokine dependent. An important cytokine for DC development is the FMS-like tyrosine kinase 3 ligand (FLT3L) [47]. Pulendran et al. found that humans injected with FLT3L showed increased numbers of pDCs, cDC1s, and cDC2s [48]. However, the differentiation into a specific subtype required specific transcription factors, particularly *IRF8* and *IRF4* [49]. The distinct DC subsets are described below in more detail and summarized in Figure 1.

### 2.2. DC Subsets

#### 2.2.1. Classical or Conventional DCs (cDCs)

cDCs, sometimes referred to as myeloid DCs, can be further subdivided into two main subpopulations termed cDC1 and cDC2. Both subsets are present in lymphoid (e.g., spleen, lymph nodes, and BM) and non-lymphoid tissues (e.g., lung and skin). In humans, the cDC1s are characterized by the low expression of CD11b and the high expression of B and T cell lymphocyte attenuator (BTLA; CD272), cell adhesion molecule 1 (CADM1; also known as NECL2), C-type-lectin CLEC9A (also known as DNGR-1), and blood dendritic cell antigen 3 (BDCA3; also known as CD141) [50,51,52]. Comparative studies revealed a corresponding cDC1 subpopulation in mice. Murine cDC1s, found in lymphoid tissues, are characterized by CD8α^+^ expression, whereas their counterpart in non-lymphoid organs features CD103^+^ expression [50,53]. In addition, mouse cDC1s can be distinguished from other DC subtypes due to high levels of Clec9A [54]. Since the cDC1 subpopulation is identified across species (mice and humans), a unifying marker, the XC chemokine receptor 1 (XCR1), has been reported [50,54]. On the other hand, cDC2s in humans are identified by the markers CD11b, SIRPα (CD172a), and BDCA1 (also known as CD1c), while the murine equivalent is generally characterized by the markers CD11b and SIRPα (CD172a) [53]. In addition to cDC1 and cDC2, migratory DCs (mig-DCs, also termed mregDC, LAMP3^+^ DC, or DC3) were recently identified as a distinct DC cluster that is present in various tumor models. These cells are mature DCs enriched in immunoregulatory molecules, and it has been suggested that both cDC1 and cDC2 can differentiate into mig-DCs [55,56,57,58].

The differentiation into specific cDC subsets is coordinated by specific transcription factors. Inhibitor of DNA binding 2 (*ID2*) [59], basic leucine zipper ATF-like 3 transcription factor (*BATF3*) [60], nuclear factor interleukin 3 regulated (*NFIL3*) [61], and the interferon regulatory factor 8 (*IRF8*) [62] are required for cDC1 specific differentiation. Upon deletion of either of these genes, the development of CD8α^+^ and CD103^+^ cDC1s was found to be defective, while CD11b^+^ cDC2 development remained unaffected. These findings suggest that the development of both the CD8α^+^ cDC1s in lymphoid tissue and the CD103^+^ cDC1s in non-lymphoid tissue depends on the same transcription factors [60]. By contrast, Seillet et al. found that these four transcription factors (*ID2*, *IRF8*, *BATF3*, and *NFIL3*) are necessary for CD103^+^ cDC1 development, while only IRF8 seems to be important for CD8α^+^ cDC1s development [63]. Furthermore, cDC2 specific differentiation depends on the transcription factors zinc finger E box–binding homeobox 2 (*ZEB2*) [64], reticuloendotheliosis viral oncogene homolog B (*RelB*) [65], PU.1 [66], recombining binding protein suppressor of hairless (*RBP-J*) [67], neurogenic locus notch homolog protein 2 (*NOTCH2*) [68], *IRF2* [69], and *IRF4* [62]. Further characterization of the murine cDC2 subsets by Brown et al. revealed two novel cDC2 subsets, termed cDC2A and cDC2B, which are defined by the transcription factors *T-bet* and *RORγt*, respectively [70]. Villani et al. described the analogy of the murine cDC2A and cDC2B subtypes with novel human blood cDC2 (CD1c^+^) subsets, termed DC2 and DC3. These human cDC2 subsets have been described as CD32B^+^CD1c^+^ cDC2 and CD163^+^CD1c^+^ cDC2, respectively [70,71]. Based on the subsequent inflammatory potential and maturation state of the human cDC2 subsets, Dutertre et al. further described three different subpopulations within the DC3 subset as follows: CD5^−^CD163^−^ cDC2s, CD5^−^CD163^+^ cDC2s, and CD5^−^CD163^+^CD14^+^ cDC2s [72].

#### 2.2.2. Plasmacytoid DCs (pDCs)

pDCs share several characteristics with cDCs, as they are both strongly FLT3L-dependent during development [47,73]. In addition, pDC development depends on specific transcriptional regulators, including *E2-2*, *Spi-B*, *IRF8*, and *ID2* in humans and mice [62,64,74,75]. pDCs are CD11c^low^, MHC-II^+^, CD317^+^, and sialic acid-binding immunoglobulin-like lectin-H (Siglec-H^+^) in mice. In human, pDCs are characterized by CD123 (IL3Rα), BDCA2 (CD303, CLEC4C), and BDCA4 (CD304) expression [40,52,76]. The unifying marker CD45RA (B220) identifies both mouse and human pDCs [46,76].

#### 2.2.3. Monocyte-Derived DCs (moDCs)

Inflammatory DCs are DCs originating from circulating blood monocytes upon inflammatory conditions that include phenotypes previously described as monocyte-derived cells, TNFα/iNOS-producing DCs (Tip-DCs), or moDCs [77]. Upon injury, CCR2 controls the exit of monocytes (Ly6C^+^) out of the BM and the consequent invasion into the inflammatory tissue. Subsequently, these infiltrated monocytes fully differentiate into moDCs in response to growth factors, such as granulocyte-macrophage colony-stimulating factor (GM-CSF) or Toll-like receptor 4 (TLR4) ligands. Similar to cDC2s, moDCs depend on the transcription factor *IRF4* for differentiation [77,78]. Furthermore, the phenotypic profile of moDCs is highly similar to that of the cDCs since both subtypes are expressing CD11c, MHC-II, and CD11b molecules on their surface. The markers C-C motif chemokine receptor 2 (CCR2) and Fc-gamma receptor 1 (FcγR1; CD64) distinguish moDCs from cDCs, as they are remnants from their monocytic past [79]. In summary, human and murine moDCs are CD11c^+^, MHC-II^+^, F4/80^−^, CCR2^+^, CD14^+^, CD64^+^ (FcγR1^+^), and CD16^+^ (FcγR3^+^) [40,80].

### 2.3. DC Biology in Healthy Tissue

DCs are master APCs that bridge the innate and the adaptive immune system and are therefore indispensable for an adequate immune response [27]. DCs arise in the BM niche in an immature state, after which they distribute in lymphoid and non-lymphoid tissues. In peripheral tissue, immature DCs (imDCs) are capable of recognizing and capturing Ags as well as tumor-(neo)antigens. As sentinel cells of the innate immune system, DCs are able to recognize endogenous danger molecules, called damage-associated molecular pattern molecules (DAMPs) that are released by damaged or dying cells, via pattern recognition receptors (PRRs) on the cell surface. Subsequently, DCs secrete the necessary cytokines allowing the activation of innate immune cells [81]. Simultaneously, DCs process and present these Ags on the cell surface, allowing imDCs to switch into mature DCs (mDCs). While imDCs are characterized by low levels of co-stimulatory molecules (CD80, CD86, and CD83) and the low secretion level of immunostimulatory cytokines (interleukin-12 (IL-12), IL-10, and tumor necrosis factor (TNF)), mDCs express high levels of co-stimulatory molecules and immunostimulatory cytokines [82]. In addition, imDCs conserve the MHC-II molecules in the late-endosomal and lysosomal compartments, whereas in mDCs, they are located on the cell surface [83]. Upon Ag presentation via MHC molecules, mDCs migrate to draining lymph nodes in a chemokine-dependent manner [84]. CCR7 and its cognate ligands, the C-C motif ligand (CCL)-19 and CCL-21, allow homing of DCs through the lymphatic vessels to the T lymphocyte-enriched zone in the secondary lymphoid organs [84,85,86]. In the draining lymph nodes, mDCs trigger naïve T cells to differentiate into diverse effector T cells, including Th1 cells, Th2 cells, Th17 cells, T follicular helper (TFH) cells, Tregs, and CD8^+^ cytotoxic T lymphocytes (CTLs), resulting in specific T cell responses [87,88]. Depending on the Ag nature, with MHC-I presenting endogenous Ags and MHC-II presenting exogenous Ags, DCs elicit adaptive CD8^+^ or CD4^+^ T cell immune responses, respectively. However, an interesting process called “cross-presentation” allows the presentation of exogenous Ags on MHC-I molecules instead of on MHC-II molecules, resulting in CD8^+^ CTL activation [89].

In more detail, each DC subset contributes differently to the immune response. For instance, cDC1s excel in cross-presenting exogenous Ags via MHC-I to CD8^+^ CTL and secrete IL-12, thereby promoting Th1 responses [49,90]. In addition, cDC1s are experts in activating NK cells and NKT cells. Both functional properties make them superior for inducing an effective anti-tumor immune response. By contrast, cDC2s are excellent activators of, preferably, CD4^+^ T cells (to a lesser extend CD8^+^ T cells), allowing the induction of Th1, Th2, and Th17 via MHC-II molecules [91]. As a result, cDCs are crucial for activating and maintaining a specific immune response, with cDC1 mainly initiating a cellular immune response and cDC2 a humoral immune response. Furthermore, functional experiments showed that the recently described cDC2 subpopulations exert distinct immune responses. The cDC2A subpopulation is involved in anti-inflammatory control, whereas the cDC2B subpopulation is found to produce TNFα and IL-6 [70]. Recently, some cDC2 subsets were also shown to cross-present Ag to CD8^+^ T cells [92,93] or further activate existing CD8^+^ T cells [94]. The human blood DC2 and DC3 subsets are shown to be potent stimulators of naïve T cell proliferation [71]. In addition, pDCs are essential for regulating an antiviral innate immune response, as their activated state is capable of secreting large amounts of interferon-α/β (IFN-α/β) upon TLR7/9 stimulation [95]; however, overproduction of IFN-α/β is associated with autoimmune disorders. Furthermore, in several cancers, pDCs are correlated with a poor prognosis, as they promote immunosuppression by the expansion and activation of Tregs [96]. In contrast to cDCs, pDCs are limited in their ability to activate naïve CD4^+^ and CD8^+^ T cells [97]. Finally, moDCs, differentiated from monocytes that were recruited to the site of injury, play a major role in inflammatory control. MoDCs have been widely tested in several clinical settings and are found to be prominent in Ag uptake and processing; however, their Ag presentation capacity, migration potential to the lymph node, and the secretion capacity of crucial cytokines (e.g., IL12p70) is less efficient [98,99,100].

Importantly, due to the capacity of DCs for Ag (cross-)presentation and the induction of specific cellular (T cells) and humoral (B cells) immune responses, DCs are a promising tool for immunotherapy. However, in literature, it has been described that DCs collected from MM patients feature defective immunological properties, supporting MM pathogenesis [20,21,101].

## 3. DC Deficiencies in Multiple Myeloma (MM)

**Figure 2:**
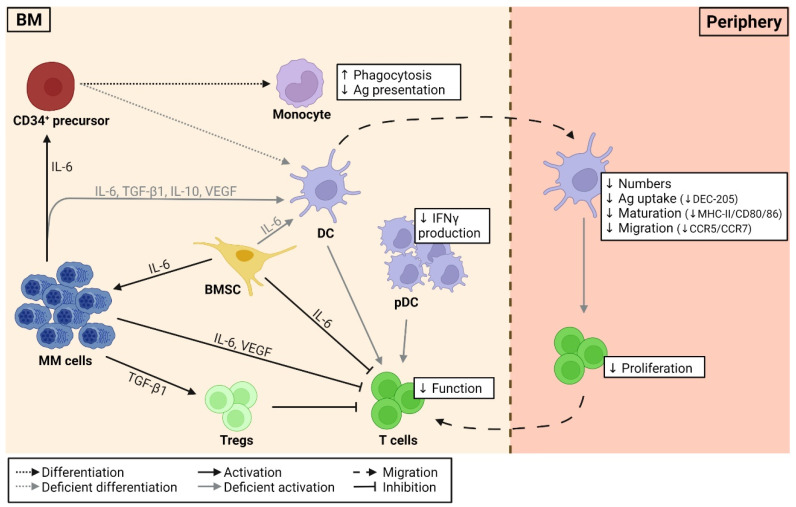
Schematic overview of the factors engaged in DC deficiencies in MM. DC differentiation, maturation, and activation are hampered by tumor microenvironmental factors. Tumor-derived cytokines, such as IL-6, promote CD34^+^ precursor cell differentiation into monocytic cells instead of DC progenitors, whereas TGF-β1, VEGF, and IL-6/-10 are found to be responsible for impaired DC maturation (decreased MHC-II/CD40/CD80/86) and function (decreased CCR5/CCR7/DEC-205). Impaired DCs lack efficient T cell activation. Tumor-derived factors, such as IL-6 and VEGF, can directly inhibit T cell function, whereas TGF-β1 favors the differentiation and expansion of Tregs, which on their terms suppress T cell function. pDCs, found in MM patients, show defective interferon (IFN)-γ production and accumulate in the BM niche, exerting immunosuppressive and tumor-promoting properties. The tumor microenvironment promotes MM cells survival through, for instance, the secretion of IL-6 by BMSCs. DC, dendritic cell; MM, multiple myeloma; BM, bone marrow; Ag, antigen; BMSCs, BM stromal cells; pDC, plasmacytoid DCs; Tregs, regulatory T cells; IL-6, interleukin 6; TGF-β1, transforming growth factor-β1; VEGF, vascular endothelial growth factor.

DC deficiencies in MM are summarized in Figure 2 and are known to play an important role in MM pathophysiology, as they lack their proper immunological capacity, resulting in drug resistance and the subsequent failure of current immunotherapeutic approaches. Despite numerous studies on DC deficiencies in MM, the presence and function of the different DC subsets, particularly the cDC subsets, remains to be elucidated. Several researchers investigated the number, phenotypic profile, and functional status of DCs during MM disease progression. The number of circulating DCs in healthy individuals includes 0.1–2.0% of the mononuclear cells [102,103]. However, a significant difference between MM patients and healthy individuals has been observed, with approximately a 50% reduction in myeloid DCs (BDCA1^+^) and pDCs (BDCA2^+^) in the peripheral blood (PB) of MM patients [20,21,101]. This reduction in PBDCs is independent of the patient’s disease stage (stage I vs. stage III MM) [20]. Regarding the BM DC number, Leone et al. found that during disease progression from MGUS to active/symptomatic MM, the myeloid DCs (CD11c^+^) and pDCs (CD11c^–^ CD123^+^) accumulate in the BM niche [104]. Moreover, this accumulation is accompanied by an increased tumor burden, as they exert immunosuppressive and tumor-promoting properties [104,105].

Besides the changes in DC numbers, significant alterations of the phenotypic profile of DCs have been observed in MM patients. CCR5, CCR7, and DEC-205 expression was down-regulated on myeloid DCs (BDCA-1^+^) and pDCs (BDCA-2^+^) in PB from MM patients when compared to those derived from healthy controls [21]. Immature DCs migrate to the site of inflammation under the guidance of CCR5, while migration of mature DCs to the secondary lymph nodes is controlled by CCR7 [21]. Consequently, reduced expression of CCR5 and CCR7 molecules in MM DCs is associated with disturbed DC migration, whereas reduced expression of DEC-205 decreases Ag uptake. In addition, the significantly lower expression of HLA-DR molecules and co-stimulatory molecules, such as CD80/86 and CD40, may indicate that PBDCs (myeloid DCs (BDCA-1^+^ or CD11c^+^ CD33^+^) and pDCs (BDCA-2^+^ or CD123^+^)) in MM patients are in an immature state and as a consequence possess impaired Ag presentation capacities [20,21]. As a result, T cell proliferation and activation are insufficient, lacking a proper immune response.

Phenotypic alterations and functional deficiencies, which include impaired DC differentiation, maturation, and activation, are influenced by immunological inhibitory cytokines present in the tumor microenvironment (Figure 2). The most commonly involved cytokines are transforming growth factor-β1 (TGF-β1), vascular endothelial growth factor (VEGF), IL-6, and IL-10. These factors can induce hyperactivation of the STAT3 and extracellular signal-regulated kinase (ERK) pathways, which may be taken accountable for defective DC differentiation [106,107]. TGF-β1 and IL-10 are both secreted by MM cells and are thought to be responsible for deficient CD80/86 upregulation during DC maturation [20,108]. Blocking these molecules with, for instance, anti-TGF-β1 or anti-IL-10 antibodies, or by administering IL-12 and IFN-γ, neutralizes the inhibition in CD80/86 upregulation [109]. In addition, the excessive production of TGF-β1 by MM cells suppressed allogeneic T cell responses and favored the differentiation and expansion of Tregs, resulting in tumor-associated immune tolerance [110]. Besides the fact that tumor-derived VEGF is engaged in the impaired DC function due to inhibitory effects on DC maturation and differentiation, it is also responsible for T cell exhaustion [111,112]. Previous research also confirmed the importance of the adhesion of MM cells to BM stromal cells (BMSCs). BMSCs secrete IL-6 in an NF-κB-dependent manner, supporting MM cell growth and survival. IL-6, which can also be secreted by MM cells, affects CD4^+^ T cell differentiation, as it inhibits Th1 polarization and promotes Th2 differentiation. Furthermore, IL-6 promotes CD34^+^ precursor cell differentiation into monocytic cells instead of DC progenitors through the upregulation of CD14 and downregulation of CD1a, HLA-DR, CD40, and CD80 [20]. As a result, these monocytic cells feature adequate phagocytosis but inadequate Ag presentation. Brimnes et al. were the first to report that pDCs (BDCA-2^+^), present in MM patients, show defective IFN-γ production, which is indispensable for regulating a proper antiviral innate immune response [21]. Wang et al. confirmed the inhibitory effect of tumor-derived IL-6, IL-10, and TGF-β1 on DC maturation and function using the 5TGM1 model. This maturation block resulted in a weakened expression of MHC molecules and co-stimulatory factors on the DC surface and an insufficient capacity of DCs to prime allogeneic T cell responses [113].

In conclusion, MM cells are considered intelligent evaders of immunosurveillance through their ability to disturb B cell immunity, promote Treg expansion, and suppress CTL activity but, most importantly, because of their negative impact on DC differentiation, maturation, and functions [114].

## 4. Current DC-Based Immunotherapies in MM

DC-based strategies aiming to elicit effective anti-MM immune responses have evolved tremendously over the past few decades. Initial trials made use of the naked idiotypic (Id) protein to immunize MM patients. However, additional attempts using Id-proteins conjugated with immunogenic carriers, such as keyhole limpet hemocyanin (KLH) or GM-CSF, resulted in unsatisfactory immune responses [2,115,116]. APCs are, because of their functional properties, suitable natural adjuvants and thus considered a promising tool for cancer immunotherapy, including vaccination therapies. Dabaghao et al. demonstrated that DCs rather than monocytes are the most potent APCs to elicit an efficient primary Id-specific immune response due to their capacity to present (tumor-) Ags via MHC molecules in the context of the necessary co-stimulatory signals [117].

Thus far, several attempts of DC-based strategies in MM have been conducted in a clinical setting. Accordingly, several DC-based vaccination “generations” have emerged over time, evolving from “first-generation” to “second-generation” and the rising “next-generation” DC vaccines. Central to the first and second generations are the use of ex-vivo-generated moDCs. Different protocols for generating a sufficient number of moDCs from human monocytes have been reviewed elsewhere [118]. In summary, adherent peripheral blood mononuclear cells (PBMC; CD14^+^ monocytes), collected through leukapheresis, were cultured in the presence of differentiation cocktails (e.g., GM-CSF, IL-4), resulting in immature moDCs (considered first-generation DC vaccines), and subsequently, maturation cocktails (TNF-α, IL-13, stem cell factor (SCF), TGF-β1, FLT3L, or CD40 ligand (CD40L)) were used to obtain mature moDCs (considered second generation) [119]. MoDCs were either loaded with Id-proteins [120,121,122,123,124,125,126,127,128,129,130,131,132,133], pulsed with myeloma-associated antigen mRNA (such as MAGE3, BCMA, and Survivin) [134], or fused with whole tumor MM cells [135,136,137]. In Table 1 and Table 2, we give an overview of the DC-based strategies that were performed in clinical trials for MM patients.

### 4.1. The Idiotypic (Id) Protein as MM-Specific Tumor-associated antigen (TAA) for DC-Based Immunotherapy

Initial clinical trials of DC-based therapies used the Id-protein as MM-specific tumor-associated antigen (TAA) in order to elicit specific immune responses in advanced-stage MM patients [120,121,126,128,131]. For instance, Wen and colleagues were the first to report DC-based immunotherapy in a 43-year-old MM patient. Prior to DC vaccination, the patient’s serum Id level was 31 g/L and quickly progressed up to 41 g/L. Although the serum Id level initially dropped to 35 g/L after the first vaccine, the Id levels increased again to 47 g/L 5 weeks after the first dose despite two additional doses. Regardless of the poor clinical responses, they were able to demonstrate the adequate functional abilities of ex-vivo-generated moDCs, including uptake, processing, and presenting Id to prime T cells [120]. In order to enhance the efficacy of DC-based therapy, boosting strategies emerged. The addition of GM-CSF could substantially improve anti-tumor immune responses by increasing T cell immunity. Cull et al. showed Th1-like immune responses, which were associated with the production of IFN-γ; however, an Id-specific CTL response was absent upon DC-based therapy and GM-CSF in advanced refractory MM patients [126]. Titzer et al. tested DC-based therapy, followed by three booster events with either Id-peptides alone or Id-pulsed DCs in combination with GM-CSF in patients with advanced MM. Results showed that three out of 10 patients possessed an adequate Id-specific humoral immune response. Four out of 10 patients demonstrated a cellular immune response. Although one patient showed decreased BM MM cell infiltration, all remaining patients showed aggravation of disease [128].

A possible explanation of the poor clinical responses might be the advanced disease stage of the patients included in previous clinical trials. Alternatively, the competence of DC-based therapy in patients with early-stage/early-relapse MM was examined by Lim and colleagues. Importantly, one patient showed a 25% reduction of the serum Id level, going from 20–21 g/L pre-vaccination to 15–17 g/L post-vaccination. In contrast to the situation in advanced-stage MM patients, where the clinical response lasted less than five weeks [120,126,128], in early-stage MM patients, the clinical response remained for over 13 months post-DC vaccination. In addition, serum Id levels of two patients remained consistent during the follow-up of 81 months. Two patients showed disease progression two and five months post-DC vaccination [127].

While previous clinical trials included early- or advanced-relapse MM patients following various chemotherapy regimens, subsequent clinical trials examined the feasibility of Id-based DC therapy in MM patients undergoing high-dose chemotherapy and auto-HSCT. For instance, in the study of Reichardt et al., two patients were in complete remission (CR), nine were in partial remission (PR), and one had stable disease (SD) following high-dose chemotherapy and auto-HSCT. After a follow-up time of minimum 16 months post-auto-HSCT, the two initial patients in CR remained in the same condition, three of the nine patients that were initially in partial remission (PR) were deceased, and the one patient in SD remained in SD. Moreover, the SD patient’s serum Id level decreased from 2.3 g/dL pre-vaccination to 1.8 g/dL post-vaccination [121,131]. In the study of Liso et al., 17 of the 26 MM patients subjected to DC-based therapy survived the follow-up time of 34 months post-auto-HSCT. Of the five patients that were in CR post-auto-HSCT, one was deceased due to PD, whereas the other four patients were still in CR. Of the 21 patients in PR post-auto-HSCT, eight were deceased, whereas seven were in PR, and two were in CR [129]. Bendandi and colleagues were the first to investigate the potential of allogeneic, ex-vivo-generated moDCs pulsed with the Id. This trial included four MM patients with relapse or progressing disease who were previously subjected to reduced intensity conditioning, allogeneic HSCT, and rescue therapy with donor lymphocyte infusion. After the DC vaccination protocol, three patients had PD, whereas only one had SD [132]. Finally, Lacy et al. compared the outcome of 27 patients who had auto-HSCT followed by Id-loaded DC vaccination (APC8020; Mylovenge™) to that of 124 consecutive patients who only received auto-HSCT during the same period. These findings suggest that auto-HSCT followed by DC vaccination improved the overall survival by almost two years when compared to the group that missed DC-based therapy [122]. Similar conclusions were drawn by Zahradova et al., as DC-based therapy prolonged the duration of SD achieved after high-dose chemotherapy and auto-HSCT in a phase II clinical trial [125].

Yi and colleagues [124,130] argued that there are several limitations concerning the DC vaccination protocol performed in previous clinical trials, including in particular the intravenous (i.v.) administration of immature moDCs [117,121,126,128,129]. Immature moDCs are at risk to differentiate into macrophages in absence of additional cytokines, and their immature state makes them less potent in activating an adequate T cell response. In addition, i.v. administration may reduce the potential of DCs to migrate to lymphoid tissues. Accordingly, Yi et al. reported that the immunological response was more effective in stable MM patients subjected to subcutaneous (s.c.) administered mature moDCs; however, one of the five participants lacked immunological responses to vaccination and showed signs of relapse [130]. Confirmed by Curti et al., s.c. administered mature DCs induced Id-specific T cell proliferation and T cell responses with high potency up to one year after DC-based therapy. In addition, they demonstrated that similar effectivity was achieved with whole Id-protein (6/15 patients) as well as with Id-VDJ-derived peptide (9/15 patients) loaded DC-based therapy [133]. An alternative injection site to improve T cell priming upon DC-based therapy is the intranodal (i.n.) route, resulting in Id-specific T and B cell responses in MM patients. Five years post-DC-based therapy, four patients remained in SD, whereas the other four patients showed PD, and one patient was lost during follow-up [124]. Another reason for the unsatisfactory immune responses could be the flawed patient selection, as the majority of the clinical trials included MM patients featuring a compromised immune system due to previous systemic treatments (e.g., high-dose chemotherapy and auto-HSCT). Röllig and colleagues were the first to report the immune effects after DC-based therapy in patients with MM clinical stage I who received no prior systemic treatment. An Id-specific T cell response was observed in 56% of the patients. Although specific cytokine secretion was reported, no sustainable Th1 or Th2 responses were detected. In addition, a slight decrease in the serum Id level was observed in three patients [123].

These findings suggest that the Id-protein, as MM-specific TAA, might be unqualified for inducing a proper immune response. The Id-protein may not be as immunogenic as initially thought due to their low expression on MM cells. Therefore, it should be noticed that the choice of TAA determines the efficacy of DC vaccination therapy.

### 4.2. MM-Associated Antigen mRNA for DC-Based Immunotherapy

As an alternative for Id, a broad range of MM-associated antigens have been identified. Several preclinical studies have demonstrated the potency of alternative MM-specific TAA for DC-based immunotherapy, including hTERT and MUC1 [138], heat-shock proteins [139], spermatozoa protein (sp) 17 [140] (clinicaltrials.gov identifier: NCT03591614), Dickkopf-1 (DKK1) [141], and the cancer-testis antigens melanoma-associated antigen (MAGE)-C1 (CT-7) [142] and NY-EOS-1 [143]. Currently, the MM-TAAs MAGE3, Survivin, and BCMA have been proven safe in a phase I clinical trial by Hobo et al. DC-based immunotherapy was based on matured moDCs pulsed with KLH and electroporated with mRNA of the TAA (*MAGE3*, *Survivin*, or *BCMA*). MM clinical stage II or III patients eligible for DC-based therapy were previously subjected to high-dose chemotherapy and auto-HSCT. Overall, TAA mRNA-loaded DC vaccination was well tolerated and was found to elicit a TAA-specific CTL response in two MM patients. After a median follow-up time of 55 months post-HSCT, 10 of the 12 patients were alive, half of whom had SD, while the other half were in PD [134]. Currently, DC loading with the Survivin antigen using an adeno-associated vector (clinicaltrials.gov identifier: NCT02851056) and DC-based therapy with Wilms Tumor 1 Gene (*WT1*) mRNA (clinicaltrials.gov identifier: NCT00965224) are being translated to the clinic for patients with MM. Moreover, an early phase I clinical trial, expected to start in October 2022, will study the safety and efficacy of DKK1 as MM-TAA in DC-based immunotherapy for MGUS, SMM and active MM patients (clinicaltrials.gov identifier: NCT03591614).

Further investigation into MM-specific TAAs that are abundantly expressed on MM cells is required. Cancer-testis antigens are promising targets for DC-based immunotherapy, as they are predominantly expressed on tumor cells and, to a lower extent, on healthy tissue [144]. However, a potential restriction of using a single TAA for DC-based therapy is the vulnerability to immune evasion. Tumor cells have the capacity to escape antitumor immunity by downregulation of Ags, lacking long-term clinical responses upon DC-based immunotherapy [145,146].

### 4.3. Total MM-Antigen Spectrum for DC-Based Immunotherapy

Loading ex-vivo-generated moDCs with the total MM-antigen spectrum can overcome the limitations associated with the use of a single TAA for DC-based immunotherapy. Such an alternative source includes the fusion of moDC with patient-derived MM tumor cells using polyethylene glycol [135,136]. This approach enables a polyvalent immune response with the opportunity to target patient-specific neoantigens. Within this context, two clinical trials by Rosenblatt et al. have been reported [135,136]. Eleven patients displayed a two-fold rise in CD4^+^ and CD8^+^ T cell quantities, associated with disease stabilization upon DC/MM cell fusion DC-based therapy [135]. A phase II clinical trial of Rosenblatt et al. reported the therapeutic use of DC fusion vaccines in 36 MM patients in total. This study was subdivided into two cohorts, with, on the one hand, 24 patients receiving auto-HSCT followed by a series of four DC/MM fusion vaccines at a four-week interval (cohort 1) and, on the other hand, 12 patients receiving one DC/MM fusion vaccine prior to auto-HSCT and four vaccinations post-transplantation (cohort 2). Although DC-based therapy post-HSCT boosted the CD4^+^ and CD8^+^ tumor-specific T cell proliferation, no difference in T cell response was observed between patients receiving DC therapy prior to auto-HSCT and those only receiving DC therapy post-HSCT [136].

Besides DC/MM tumor cell fusion vaccines, loading DCs with tumor cell apoptotic bodies, tumor cell lysates, or total tumor RNA can provide the total MM-antigen spectrum for DC-based immunotherapy. For instance, Vasileiou and colleagues loaded ex-vivo-generated moDCs by performing two different protocols [147]. The first protocol included the uptake of apoptotic bodies from gamma-irradiated autologous myeloma cells (AMC) through phagocytosis, while in the second protocol, AMC total RNA was transfected by square-wave electroporation. In-vitro analysis showed that both protocols are efficient in priming a proper MM-specific immune response, as both CD4^+^ T cells and CD8^+^ CTL were expanded in numbers upon co-culture with the loaded moDCs. Although findings were mostly similar, data suggest that the RNA transfection protocol allows a higher number of antigens to be processed through the endogenous pathway instead of being cross-presented. These in-vitro results demonstrate that the use of whole tumor cell-loaded moDCs holds promise for translation into the clinic [147]. The safety and immunological efficacy of moDCs loaded with ultraviolet B-irradiated autologous dying MM cells (VAX-DC therapy) in patients with relapsed or refractory MM was reported by Jung and colleagues (clinicaltrials.gov identifier: NCT02248402) [137]. Although VAX-DC therapy favors the capturing and presentation of multiple epitopes (including potential unidentified TAAs) as well as the broad spectrum of T cell responses, the harvesting of a sufficient amount of MM cells is restricted due to previous treatments. All patients were alive after a follow-up time of 16.1 months; however, eight patients showed PD, of whom five received additional therapy [137].

**Table 1:** Overview of clinical trials using DC-based vaccination strategies in MM patients.

Year	Number of Participants	Stage of Disease	Prior Treatment	DC Source	Tumor Antigen	Antigen Loading	DC Number	Site of Injection	DC-Vaccination Protocol	Boosting Strategy	Ref.
Id-loaded moDCs for DC-based immunotherapy											
1998	*n* = 1	Advanced-stage refractory MM	Various chemotherapy regimens	PBMCs (adherent cells)	Tumor Id-proteins	Ex-vivo-generated immature moDCs were loaded with Id and the control vaccine with KLH	3 doses with 5 × 10^6^, 30 × 10^6^ and 45 × 10^6^, respectively	i.v.	3 doses at a 2-week interval	No boosting strategy	[120]
1999	*n* = 14 (2 declined to participate)	MM clinical stage III	High-dose chemotherapy (e.g., melphalan) and auto-HSCT	PBMCs (precursor DCs)	Tumor Id-proteins	Ex-vivo-generated immature moDCs were loaded with Id	5.1 × 10^6^ ± 2.9 × 10^6^	i.v.	2 doses at a 4-week interval	5 s.c. boosts of Id/KLH with adjuvant, administered 4 weeks after the second DC vaccine at a 4-week interval	[121]
1999	*n* = 2	Progressive refractory MM	Chemotherapeutic regimes and auto-HSCT, plus (only for P1) monthly i.v. pamidronate treatment (continuing during trial)	PBMCs (adherent cells)	Tumor Id-proteins	Ex-vivo-generated immature moDCs were loaded with Id and KLH	P1: 2 times 4 × 10^6^ and 2 times 2.5 × 10^7^ P2: 2 times 2.5 × 10^7^ and 2 times 4 × 10^7^	NS	4 doses at a 2-week interval	DC vaccination followed by s.c. GM-CSF	[126]
1999	*n* = 6	Early-stage/early-relapse MM	3/6 patients had chemotherapy and dexamethasone	PBMCs (adherent cells)	Tumor Id-proteins	Ex-vivo-generated immature moDCs were loaded with Id and/or KLH	3.5 × 10^6^–89 × 10^6^	i.v.	5 patients had 3 doses, 1 patient (P002) 2 doses	Each vaccine was supported by i.v. chlorpheniramine treatment	[127]
2000	*n* = 12 (*n* = 10 were vaccinated)	MM clinical stage II and III	Chemotherapy	CD34+ stem cells	Tumor Id-proteins	Ex-vivo-generated immature moDCs were loaded with Id	1 × 10^6^–4 × 10^7^	s.c.	1 dose	3 boosts of Id-proteins and GM-CSF (9/11) or with Id-loaded DCs (2/11)	[128]
2000	*n* = 26	MM clinical stage IIA	High-dose chemotherapy and auto-HSCT	PBMCs	Tumor Id-proteins	DCs were cultured with either Id or with Id-KLH conjugates	First 12 patients: 3.0–19.1 × 10^6^ Other 14 patients: 21.1–511.7 × 10^6^	i.v.	12 patients received 2 doses of Id-loaded DCs; 14 patients received 2 doses of DCs loaded with Id/KLH	5 s.c. boosts of Id-KLH conjugates at a 4-week interval	[129]
2002	*n* = 5	Patients are stable or in partial remission	High-dose chemotherapy and auto-HSCT	PBMCs (adherent cells)	Tumor Id-proteins	Ex-vivo-generated immature moDCs were loaded with Id and then matured	20 × 10^7^	s.c.	3 doses at a 2-week interval	Low-dose of recombinant IL-2 was given s.c. for 5 days following each vaccination	[130]
2003	*n* = 12	MM clinical stage II and III	High-dose chemotherapy and auto-HSCT	PBMCs (adherent cells)	Tumor Id-proteins	Ex-vivo-generated immature moDCs were loaded with Id and then matured	Median of 4.5 × 10^6^ DCs were obtained	i.v.	2 doses: at day 0 and day 15	10/12 patients had 5 s.c. Id/KLH booster immunizations (at a 4-week interval) co-injected with GM-CSF (for 3 consecutive days)	[131]
2006	*n* = 4	Patients with relapse or progressing disease	Reduced intensity conditioning, allogeneic HSCT, and rescue therapy with donor lymphocyte infusion	PBMCs (monocytes, (CD14 + ))	Tumor Id-proteins	Allogeneic ex-vivo-generated moDCs loaded with Id (maturation state of DCs: NS)	5–10 × 10^8^	Intra-dermal	3 cycles, each with 3 doses. Cycle 1: 3 doses at a 4-week interval Cycle 2: 3 doses at a 8-week interval Cycle 3: 3 doses at a 12-week interval	Each dose was accompanied by the s.c. administration of Id-proteins conjugated with KLH in combination with GM-CSF	[132]
2007	*n* = 15	MM clinical stage IA (8/15), IIA (2/15), IIIA (5/15)	High-dose chemotherapy, followed by auto-HSCT and maintenance therapy	PBMCs (monocytes, (CD14+))	Tumor VDJ-derived peptides or whole protein	Ex-vivo-generated immature moDCs were loaded with VDJ-derived peptides (9/15) or with whole protein (6/15) and KLH and then matured	3 s.c. doses: 5 × 10^6^, 10 × 10^6^, and 50 × 10^6^ 2 i.v. doses: 10 × 10^6^ and 50 × 10^6^	s.c./i.v.	3 s.c. doses and 2 i.v. doses at a 2-week interval	3 patients received additional s.c. injections of 50 × 10^6^ DCs, monthly, in case of stable disease and DC availability	[133]
2009	*n* = 27 (vaccine trial) *n* = 124 (database)	MM clinical stage II: 8/27 and 33/124 MM clinical stage III: 19/27 and 91/124	auto-HSCT	PBMCs (DCs and DC precursors)	Tumor Id-proteins	Ex-vivo DC precursors were co-cultured with the patient’s serum as a source for Id (APC8020 (Mylovenge™))	NS	i.v.	4 doses: given at week 0, 2, 4, and 16	No boosting strategy	[122]
2010	*n* = 9	SMM (8/9) and SD post auto-HSCT (1/9)	No prior treatment (8/9) and auto-HSCT (1/9)	PBMC (adherent cells)	Tumor Id-proteins	Ex-vivo-generated immature moDCs were loaded with Id and KLH and then matured	Median 11–13.5 × 10^6^	i.n.	4 doses at a 1-week interval	S.c. recombinant IL-2 for 5 consecutive days following each DC vaccination	[124]
2011	*n* = 9	MM clinical stage I	5 patients had bisphosphonates, and 3 had localized radiation	PBMC (adherent cells)	Tumor Id-proteins	Ex-vivo-generated immature moDCs were loaded with Id and KLH and then matured	Median 6.9 × 10^6^ (range 2.1–20.7 × 10^6^)	i.v. (5/9) or s.c. (4/9)	5 doses at a 4-week interval	No boosting strategy	[123]
2012	*n* = 25 (11/25 participants were vaccinated, 13/25 were in the control group, 1/25 was excluded)	MM clinical stage I: 4/11 and 4/13 stage II: 3/11 and 3/13 stage III: 4/11 and 6/13	Vaccinated group: auto-HSCT (9/11), 2 auto-HSCT (1/11), vaccination with Id-proteins coupled with KLH (3/11)	PBMC	Tumor Id-proteins	Ex-vivo-generated immature moDCs were loaded with Id and then matured	Median of 6.4 × 10^6^ DCs were obtained	Intradermal	6 doses at a 4-week interval	No boosting strategy	[125]
MM-TAA mRNA-loaded moDCs for DC-based immunotherapy											
2013	*n* = 12	MM clinical stage II and III	High-dose chemotherapy (e.g., melphalan) and auto-HSCT	PBMC (adherent cells)	TAA-mRNA (MAGE3, Survivin or BCMA)	Ex-vivo-generated immature moDCs were loaded with KLH and electroporated with TAA-mRNA and then matured	5–23 × 10^6^ (i.v.) 8–12 × 10^6^ (Intradermal)	i.v./ Intradermal	3 doses at a 2-week interval (P1 and P3 were revaccinated)	No boosting strategy	[134]
Total MM-antigen spectrum-loaded moDCs for DC-based immunotherapy											
2011	*n* = 18 (1 patient was excluded due to inadequate cell yields for vaccination)	All patients show signs of active disease, 2/18 patients have MM clinical stage I	14/18 had high-dose chemotherapy and auto-HSCT 2/18 received no prior therapy	PBMC (adherent cells)	Tumor MM cells	Ex-vivo-generated immature moDCs were co-cultured with MM tumor cells at a 3:1 to 10:1 ratio and then matured	1 × 10^6^–4 × 10^6^ fusion cells	s.c.	3 doses at a 3-week interval	DC vaccination was accompanied by s.c. boosts of GM-CSF administered at the vaccination site for 4 consecutive days	[135]
2013	Cohort 1 *n* = 26 (24/26 were vaccinated)	No clinical stage specified, patients had a median of 55% plasma cells in BM at enrollment	24 patients had bortezomib-based regimen, 7 lenalidomide-based regimen, 11 thalidomide-based regimen, and 11 lenalidomide-, bortezomib-, and dexamethasone-based regimen	PBMC (adherent cells)	Tumor MM cells	Ex-vivo-generated immature moDCs were co-cultured with MM tumor cells and then matured	3.6 × 10^6^ fusion cells	s.c.	3 doses post-auto-HSCT at a 4-week interval	DC vaccination was accompanied by s.c. boosts of GM-CSF administered at the vaccine site for 4 consecutive days	[136]
	Cohort 2 *n* = 19 (12/19 were vaccinated)								1 dose prior to stem cell mobilization; 3 doses post-auto-HSCT at a 4-week interval		
2017	*n* = 16 (*n* = 12 were vaccinated)	MM clinical stage I (2/12) MM clinical stage II (5/12) MM clinical stage III (5/12)	All patients had thalidomide and bortezomib therapy. 9 patients had high-dose chemotherapy and auto-HSCT; 2 had tandem auto-HSCT	PBMC (adherent cells)	UVB irradiated tumor MM cells	VAX-DC/MM: immature moDCs were loaded with UVB irradiated dying autologous MM cells and KLH and then matured	5 × 10^6^ or 10 × 10^6^	Intradermal	4 doses at a 1-week interval	3 days prior to VAX-DC/MM injection, cyclophosphamide was i.v. administered	[137]

DC, dendritic cell; MM, multiple myeloma; PBMCs, peripheral blood mononuclear cells; Id, idiotypic proteins; i.v., intravenous; s.c, subcutaneous; i.n., intranodal; auto-HSCT, autologous hematopoietic stem-cell transplantation; P, patient; KLH, keyhole limpet hemocyanin; GM-CSF, granulocyte-macrophage colony-stimulating factor; SMM, smoldering MM; SD, stable disease; TAA, tumor-associated-antigens; MAGE3, melanoma-associated antigen 3; BCMA, B-cell maturation antigen; UVB, ultraviolet B; NS, not specified.

In general, first- and second-generation DC vaccines are proven to be safe and feasible in MM patients, independent of the patient’s clinical stage and the DC vaccination strategy. Although DC-based therapy initially provides anti-tumor immunity, long-term clinical responses are limited. Yet, a number of patients showed mild fever post-DC-based therapy. Importantly, large-scale ex-vivo moDC generation is a time-consuming and expensive process. Furthermore, ex-vivo DC differentiation requires strict compliance of the Good Manufacturing Practice (GMP) protocols and quality assurance measures [148]. Moreover, the researchers also pointed out the need for combination approaches, such as DC-based therapy accompanied by immunomodulatory agents and checkpoint inhibitors to enhance the therapeutic efficacy in MM patients. Therefore, newly improved DC-based approaches and alternative DC sources should be considered to eradicate minimal residual disease and prevent relapse of myeloma patients.

## 5. Opportunities and Future Directions for DC-Based Immunotherapy in MM

### 5.1. Combination Strategies to Enhance the Efficacy of DC-Based Immunotherapy in MM

To pursue the maximum potential of DC-based therapies, increasing attention is being paid to combining DC-based immunotherapies with conventional therapies, including chemotherapy, immunomodulatory drugs (IMiDs; lenalidomide and pomalidomide) and immune checkpoint inhibitors (ICIs). Combination strategies optimizing the efficacy of DC-based therapy, mainly in solid tumors, have been reviewed extensively elsewhere. In summary, promising combination therapies with chemotherapy, radiotherapy, and ICIs are under investigation in several (pre)clinical cancer models [94,149]. Here, the main focus concerns combination therapies for MM, as summarized in Table 2.

MM features a suppressive tumor microenvironment that fosters tumor growth, immune escape, and drug resistance [22,23]. Immune-suppressive cells, such as Tregs, TAMs, and MDSCs, and immune inhibitory pathways, including the programmed death-1 (PD-1)/programmed death-ligand 1 (PD-L1) pathway and the cytotoxic T lymphocyte-associated protein 4 (CTLA-4) pathway, could hamper DC-based immunotherapy, causing insufficient clinical responses in MM [150]. Therefore, combination strategies, including agents targeting the immunosuppressive microenvironment, may strongly improve clinical responses to DC-based immunotherapy in patients with MM.

Given that the inhibitory signals PD-L1 and PD-1 are strongly expressed on ex-vivo-generated moDCs fused with autologous MM cells and on CD4+ and CD8+ T cells derived from patients with advanced MM, respectively, this may be a reason for the unsatisfactory immune responses after DC/MM fusion vaccination as previously described [151]. PD-L1 is associated with a dysfunctional T cell phenotype and may potentially contribute to Treg inflation, disrupting the activation of an adequate T cell immune response following the DC/MM fusion vaccine [150]. Rosenblatt et al. blocked PD-1 signaling using the anti-PD-1 antibody CT-011 in a preclinical MM mouse model, showing improved activated T cell responses following DC/MM fusion vaccination [151]. Two clinical trials that are currently ongoing and combine DC vaccination and PD-1 blockade are (1) a clinical trial including MM patients undergoing serial infusions of pidilizumab (CT-011) in conjunction with DC/MM fusion vaccination following auto-HSCT (clinicaltrials.gov identifier: NCT01067287) and (2) a phase II clinical trial subjecting relapsed MM patients to DC/MM fusion vaccination in combination with another PD-1 blocker, nivolumab (clinicaltrials.gov identifier: NCT03782064) (Table 2). Luptakova et al. indicated the potential of the immunomodulating drug, lenalidomide, to support DC/MM fusion vaccination, as it boosts T cell proliferation and downgrades inhibitory factors, such as Tregs and PD-1 expression [152]. Furthermore, preclinical MM mouse models subjected to dying MM cell-loaded DC therapy in combination with anti-PD-1 alone, lenalidomide alone, or lenalidomide joined by PD-1 blockade strongly improved DC-induced immune responses when compared to solo-DC-based therapy [153]. Moreover, prolonged survival was observed after triple combination therapy (DC therapy, lenalidomide, and anti-PD-1) in the murine MM model. Similar results were obtained when combining dying MM cell-loaded DC therapy with pomalidomide and dexamethasone [154]. The most promising prognosis in a preclinical MM model was obtained after quadruple combination therapy, including dying MM cell-loaded DC therapy, pomalidomide, dexamethasone, and anti-PD-1 therapy [155]. In this regard, a phase II clinical trial is currently being performed, examining the therapeutic potential of combining lenalidomide and DC/MM fusion vaccination in MM patients previously subjected to auto-HSCT (clinicaltrials.gov identifier: NCT02728102) (Table 2). In summary, DC-based therapies supported by ICIs or IMiDs may increase the anti-MM immunity by suppressing immunosuppressive cells and inhibitory signals and activating effector cells.

### 5.2. A New Upcoming DC Source for DC-Based Immunotherapy

In MM, clinical trials have been conducted with first-generation and second-generation DC vaccines, both depending on MM patient-derived ex-vivo-generated moDCs. The compromised functionality of ex-vivo-generated moDCs is put forward as one of the main reasons for their unsatisfactory therapeutic efficacy, as sufficient immune responses cannot be achieved in clinical trials [20,21,109]. Therefore, evaluating the phenotypical and functional characteristics of these DCs is fundamental to identify the factors contributing to their limited success. Shinde and colleagues generated moDCs from MM patients samples (MM-DCs) and examined them in parallel with moDCs generated from healthy donor samples (HD-DCs) [100]. MM samples contained a less pronounced precursor population (adherent mononuclear cells; CD14^+^ monocytes), which indicates a significantly lower moDC yield when compared to HD samples. However, both MM-DCs and HD-DCs showed a mature phenotype and showed equivalent Ag uptake and allogeneic T cell activation. By contrast, the migratory capacity to the lymph node, and the secretion capacity of crucial cytokines (e.g., IL12p70) were less pronounced in moDCs derived from MM patients, making MM-DCs not as potent in inducing an anti-MM response as HD-DC. Another explanation for defective DCs in MM patients could be the immunosuppressive activity of MM cells and the suppressive tumor microenvironment as cited earlier in this review.

**Table 2:** Overview of clinical trials using DC-based combination strategies in MM patients.

Study Start Year	NCT Number	Clinical Trial	Number of Participants	Prior Treatment	Tumor Antigen	DC-Based Vaccination Strategy	Combination Therapy	Treatment Protocol
2010	NCT01067287	Phase II	*n* = 35	Auto-HSCT	Tumor MM cells	DC/MM fusion vaccine	The monoclonal antibody; CT-011	3 doses of CT-011 at a 6-week interval, starting 1–3 months following auto-HSCT DC/MM fusion vaccine was given 1 week following each infusion of CT-011
2016	NCT02728102	Phase II	*n* = 203	High-dose chemotherapy (e.g., melphalan) and auto-HSCT	Tumor MM cells	DC/MM fusion vaccine	The IMiD; lenalidomide (Revlimid)	DC/MM fusion vaccine was given on day 1 of cycles 2, 3, and 4 of lenalidomide maintenance therapy, starting 90–100 days after auto-HSCT, and continued for 2 years. GM-CSF was given daily for a total of 4 days of each cycle. One cycle lasted 28 days.
2019	NCT03782064	Phase II	*n* = 5	IMiDs and PI	Tumor MM cells	DC/MM fusion vaccine	The monoclonal antibody; nivolumab	Nivolumab was given at a 2-week interval DC/MM fusion vaccine/GM-CSF was administered 4 days per cycle

DC, dendritic cell; MM, multiple myeloma; auto-HSCT, autologous hematopoietic stem-cell transplantation; GM-CSF, granulocyte-macrophage colony-stimulating factor; IMiDs, immunomodulatory drugs; PI, proteasome inhibitor.

These findings suggest that ex-vivo-generated moDCs derived from MM patients are not the most convenient DC source for DC-based immunotherapy. Alternative sources are being investigated, including the differentiation of DCs from human pluripotent stem cells (hPSCs) [119,156]. Shinde et al. compared stem cell-derived DCs from MM patients (MM-SC-DCs) with those from healthy individuals (HD-SC-DCs) [157]. Besides a similar SC-DC yield, both MM-SC-DCs and HD-SC-DCs exhibited a mature DC cell-surface phenotype. In contrast to moDCs generated from MM samples with impaired migration capabilities and CCR7 expression, SC-DCs showed efficient migration abilities towards CCL-19. This may be due to the fact that DCs generated from SCs, regardless of HD or MM samples, adequately express CCR7 and feature a lower autocrine IL-6 secretion. This study implies the use of HSC as a source for large numbers of functional DCs. In addition, great attention has been given to the in-depth characterization of tissue-derived DC subsets in cancer, in particular the cDC subset(s), aiming to identify additional DC sources with superior features for DC-based immunotherapy. Based on these findings, a new DC source for DC-based immunotherapy is on the rise to kick-start the development of next-generation DC vaccines. Naturally occurring, tissue-derived DCs pose two major advantages over ex-vivo-generated moDCs, including superior functionality and reduced culturing time and costs [145]. Previous findings of our research group showed that both cDC1 and cDC2 subsets were able to elicit therapeutically relevant immune responses in cancer [98]. Mice vaccinated with tumor-derived cDC1s could efficiently activate CD8^+^ T cells and confer protection in tumor models in which immunosuppressive cells are present in very low numbers. Importantly, cDC2 vaccination could efficiently activate Th17 cells and led to reduced tumor growth in tumors with a strong immunosuppressive immune compartment. Moreover, next-generation DC vaccines using the cDC2 (CD1c^+^/BDCA1^+^) subset have been proven safe and feasible, with promising clinical responses, indicating the ability of combined modality treatment in patients with advanced-stage metastatic prostate cancer and patients with stage IV metastatic melanoma [158,159].

### 5.3. Timing of DC-Based Immunotherapy

The majority of the clinical trials examining the therapeutic efficacy of first- and second-generation DC vaccines have been performed in heavily treated, advanced-stage MM patients. There are studies arguing that the limited therapeutic efficacy of DC-based therapy is due to the compromised immune system, which is found in refractory and relapsed MM patients previously treated with systemic therapies [123]. High-dose chemotherapy and auto-HSCT allow fast hematopoietic recovery and prolong the period of remission compared to standard chemotherapy. Unfortunately, complete remission and total eradication of the disease is rarely achieved. Therefore, DC-based therapy and adoptive T cell therapy in those lymphopenic MM patients may be an added value to eradicate minimal residual disease and restore the patient’s immune competence [160]. The period of post-transplant lymphopoietic reconstitution is associated with enhanced responsiveness to cancer vaccines due to the depletion of inhibitory cell types, such as Tregs, that mediate tumor-associated tolerance. Furthermore, DC-based immunotherapy supported by the IMiD lenalidomide that targets the tumor microenvironment has been shown to improve clinical outcomes in MM patients who may or may not have been previously subjected to auto-HSCT [151,152]. Therefore, DC-based therapy is a particularly attractive option for MM patients undergoing auto-HSCT or patients in remission to specifically eliminate residual cancer cells and protect against relapses.

## 6. Concluding Remarks

DC-based immunotherapy has been proven to be a safe and tolerable therapeutic approach with the potential to induce clinical responses in MM patients. However, thus far, clinical responses have been inconsistent in clinical trials using first- and second-generation DC vaccines. In this regard, the limited success may partially be due to the used DC source, as ex-vivo-generated moDCs have limited functional abilities. Another explanation is related to the strong immunodeficient signature of MM patients, as both the MM cells and the tumor microenvironment can hamper proper DC differentiation, maturation, and activation. Advanced perspectives in DC biology allow the rise of new potential DC sources. For instance, tissue-derived DC subsets with superior features for DC-based immunotherapy can provide the foundation for next-generation DC vaccination in MM. Most importantly, combining DC-based therapy with therapies targeting the suppressive microenvironment (e.g., IMiDs and ICIs) as well as other therapies, such as chemotherapy, aims to improve the quality of anti-tumor immune responses and the clinical outcome in patients with MM. This is especially true for early-stage MM patients or MM patients in partial or complete remission, where treatment with DC-based immunotherapy can provide sustained remission and prevent or delay relapse.

## Data Availability

The data presented in this study are available in the article and original works.
